# Interventional factors influencing natural killer cell immunity in colorectal cancer: a systematic review

**DOI:** 10.1007/s00262-024-03900-5

**Published:** 2025-02-01

**Authors:** Wai Sinn Soh, Grace L. Burns, Tharindu Senanayake, Raquel Cameron, Kerith Duncanson, Guy D. Eslick, Sharmila S. Prasad, Stephen R. Smith, Simon Keely

**Affiliations:** 1 School of Biomedical Sciences and Pharmacy, College of Health, Medicine and Wellbeing, The University of Newcastle, Callaghan, Newcastle, NSW Australia; 2 NHMRC Centre of Research Excellence in Digestive Health, The University of Newcastle, Callaghan, Newcastle, NSW Australia; 3 Immune Health Research Program, Hunter Medical Research Institute, New Lambton Heights, Newcastle, NSW Australia; 4 School of Medicine and Public Health, College of Health, Medicine and Wellbeing, The University of Newcastle, Callaghan, Newcastle, NSW Australia; 5 Hunter Surgical Clinical Research Unit, Surgical Service, John Hunter Hospital, New Lambton Heights, Newcastle, NSW Australia

**Keywords:** Colorectal cancer, Natural killer cells, Perioperative intervention, Systematic review

## Abstract

**Background and aim:**

Colorectal cancer (CRC) is the third most common cancer globally. Despite advances in curative CRC surgery, metastatic disease remains a significant problem. Emerging evidence suggests that several commonly used perioperative interventions may influence cancer immunity through natural killer (NK) cell function or numbers. This systematic review aimed to identify the effects of standard perioperative interventions on NK cells in CRC patients.

**Methods:**

A systematic search of four databases, MEDLINE, EMBASE, Cochrane Library and Scopus was performed up to 30th July 2024 using keywords associated with colorectal cancer, NK cells and perioperative medications. PRISMA guidelines were followed. Critical appraisal was undertaken using the Joanna Briggs Institute Critical Appraisal tools for quasi-experimental studies and randomised controlled trials.

**Results:**

The literature search identified 10,404 titles and abstracts for screening. Of these, 226 full-text papers were reviewed, and 16 studies were included for data extraction. Results were presented in a narrative synthesis due to study heterogeneity. *In vivo* studies consistently demonstrated a post-operative decrease in overall cytotoxic capacity and number of NK cells compared to pre-operative levels. This effect was observed across all examined medications, including fentanyl, morphine, propofol and oxycodone. Additionally, blood transfusion also resulted in decreased NK cell cytotoxicity and activity in CRC patients.

**Conclusion:**

While surgical stress is one of the significant factor influencing NK cell cytotoxicity, this review indicates that perioperative intervention also contributes to decreased NK cell cytotoxicity. Our findings demonstrate that certain perioperative interventions (such as ketamine and morphine) can negatively impact NK cell cytotoxicity.

**Supplementary Information:**

The online version contains supplementary material available at 10.1007/s00262-024-03900-5.

## Introduction

Colorectal cancer (CRC) is the third most common cancer, with the global burden projected to increase to 3.2 million new cases and 1.1 million deaths a year by 2040 [1]. Current treatments for CRC patients include surgery, radiation therapy, chemotherapy and immunotherapy [2], but surgery remains the mainstay of treatment. Unfortunately, almost half of those who undergo resection with curative intent will subsequently experience cancer recurrence and/or metastatic disease [3]. The causes of post-surgical cancer recurrence are unknown, while the mechanisms involved are likely complex and multifactorial and are incompletely understood [4]. It is generally thought that recurrence occurs due to the proliferation of residual cancer cells [5]. However, emerging understanding of how environmental factors such as surgical stress may lead to metabolic and neuroendocrine changes suggests the post-surgical environment may facilitate the survival and implantation of circulating tumour cells into tissues, contributing to recurrence and metastatic disease [6].

Perioperative factors, such as anaesthetic medication and post-surgical opioid usage, may also influence CRC recurrence and the development of metastases via modulation of the immune system [7]. For instance, opioid use has been shown to release immunosuppressive biological amines, which inhibit the immune system by acting on opioid receptors [8]. This inhibition may contribute to the immune evasion of tumour cells by impeding the function of natural killer (NK) cells, T cells, dendritic cells and macrophages. NK cells are a critical component of the innate immune system [9] and are classified as group 1 innate lymphoid cells, which are chiefly responsible for eliminating malignant or infected cells [10]. NK cells represent 5–15% of human peripheral blood mononuclear cells (PBMCs) and are present under normal physiological conditions in the gut and other organs such as kidneys, liver, lungs, breasts and skin [11]. NK cell function and activity have long been linked with the survival of CRC patients. A low degree of NK cell infiltration or impaired NK cell function is associated with poor overall patient survival and CRC recurrence [12]. It has also been demonstrated that there is a statistically significant inverse correlation between NK cell cytotoxicity and CRC incidence in high-risk populations [13]. Moreover, enhanced NK cell activity is also associated with improved overall survival rates among CRC patients [14]. Given that NK cells play a crucial role in cancer immunosurveillance [15], this systematic review aimed to characterise how commonly used perioperative medications influence NK cell function.

## Methods

### Study protocol

A systematic search was conducted up to 30th July 2024 using the MEDLINE (OVID), Embase (OVID), Cochrane Library (Wiley Online) and Scopus (Elsevier) databases to identify relevant articles with the assistance of experienced research librarians (AS) using combination of medical subject headings (MeSH) and synonym relevant to this review. The terms used are listed in Supplementary Table 1. The PRISMA 2020 checklist was followed in this systematic review (Supplementary Table 2) [16]. This review was registered under the PROSPERO systematic review database registration, CRD42022295814.

### Study selection

Specific inclusion and exclusion criteria were used to identify all articles to be reviewed. The inclusion criteria were: i) control and treatment patients over 18 years old; ii) studies assessing the effects of pharmaceuticals in the ex vivo and/or in vivo setting; iii) studies that examine NK cell number and/or function; iv) medications were used perioperatively, or patient received blood transfusion perioperatively. Exclusion criteria were: i) non-English language manuscripts, ii) non-human studies and iii) patients who received chemotherapy agents, immunotherapy and any vaccine-based cancer therapy after the surgery. Included papers were uploaded into Covidence (Veritas Health Innovation, Melbourne, Australia) for screening, and duplicates were removed. Two authors (WSS and TS or KD) performed screening of titles and abstracts, with conflicts resolved by consensus. Full texts were uploaded and screened using the same criteria.

### Quality assessment

Study quality was assessed using either the Joanna Briggs Institute (JBI) randomised control trials (RCT) or quasi-experimental studies critical appraisal tool, depending on study design [17]. Two reviewers (WSS and RC) evaluated each study using a checklist with 13 questions (1 point each) for the RCT (Supplementary Table 3) [18] and 9 questions (1 point each) for the quasi-experimental studies tool [19]. A third reviewer (KD) resolved conflicts on the 12.5% of criteria where there was disagreement between the reviewers.

### Data extraction

Data extraction was performed using the predefining information of interest: publication year, country of origin, study design, sample size (including number of controls and cases), type and dose of medications given, colorectal cancer site, surgical approach (laparoscopic or open), NK cell detection/analysis methods and NK cell quantification. Authors were not contacted for missing data.

## Results

### Study characteristics

**Fig. 1:**
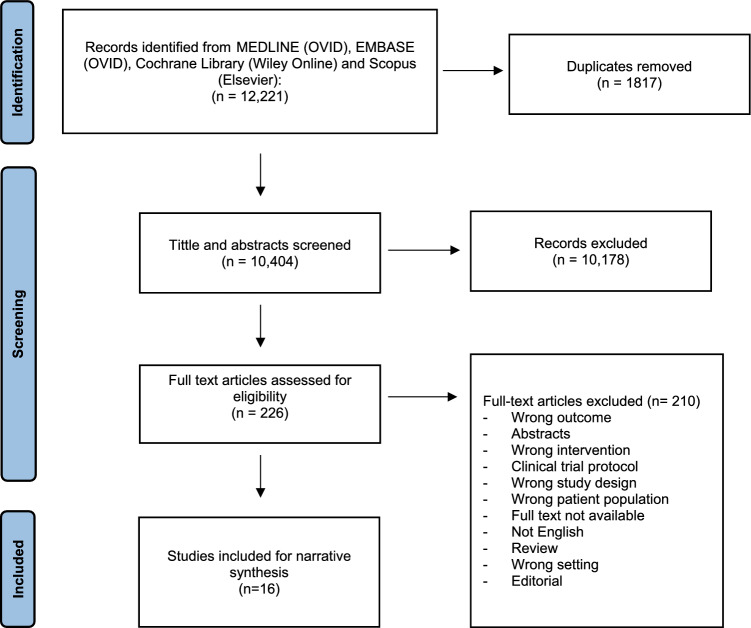
PRISMA flow diagram for the identification of studies suitable for inclusion in systematic review on perioperative intervention in colorectal cancer surgery

Of the 12,221 tittles identified in the search, 1,817 were duplicates. The remaining 10,404 titles and abstracts were screened, with 226 studies selected for full-text screening. Subsequently, 210 studies were excluded as they did not meet the study criteria for reasons due to incorrect study design, population, intervention, outcome and setting. As a result, 16 studies [20–35] were included for data extraction (Fig. 1). Given significant study heterogeneity and unavailability of raw data for meta-analysis, these studies were synthesised using a narrative approach.

### Quality assessment

All the studies met at least 60% (6/9 and 9/13, respectively) of the JBI quasi-experimental tools and RCT trial quality criteria (Supplementary Table 3). Eight quasi-experimental studies [20, 23, 24, 26, 28–30, 35] did not meet the follow-up criteria question, and two RCTs [31, 33] were “not applicable” for the criteria relating to participant blinding to treatment assignment and those who deliver treatment being blinded to the treatment assignment.

### Patient characteristics

Of the 16 included studies, four studies [21, 22, 25, 27] **(**Table 1**)** stated the clinical stage of cancer for the patients who underwent surgery. Within these studies, there were a total of 138 stage I, 161 stage II and 128 stage III cancer patients. Only two of the sixteen studies [21, 33] reported the anatomical site of the cancer.

**Table 1:** Summary data of blood collection timepoints, type of operation, measurement and assessment of natural killer cells in studies investigating perioperative interventions during colorectal cancer surgery

First author	Patient age (mean ± SD; median)	Blood collection timepoints	Diagnosis (n)	Cancer stage (n)	Site of cancer (n)	Type of operation (n)	Measurement of NK cells	Assessment of NK cells
Li et al. [20]	-		-	-	-	-	Flow cytometry	NK cell number
Oh et al. [21]	Propofol group = 62.2 ± 9.8 sevoflurane group = 61.9 ± 11	T1. Before anaesthetic T2. On arrival in the post-anaesthetic care unit T3. 24 h post-operative	Colorectal cancer (153)	Stage 0 (4), Stage I (45), Stage II (44), Stage III (46), Stage IV (14)	Ascending (25), transverse (5), descending (3), rectal (77), sigmoid (43)	Hemicolectomy (30), transverse colectomy (5), low anterior resection (76), anterior resection (26), abdominoperineal resection (16)	Flow cytometry	NK cell number
Cho et al. [22]	Control group = 61 (58.9–63.0) Ketamine group = 57.2 (53.9–60.4)	T1. Preoperatively T2. 1 h post-operative T3. 24 h post-operative T4.48 h post-operative	Colon cancer (69), rectal cancer (31)	Stage I (26), Stage II (34), Stage III (40)	-	Right-sided hemicolectomy (29), transverse colonic resection (4), left-sided hemicolectomy (6), sigmoid resection (26), low anterior resection (25)	NK Vue Kit	NK cell cytotoxicity
Song et al. [23]	Control group = 57.36 ± 4.49 Observation group = 58.13 ± 4.52(87)	T1. 30 min before anaesthesia T2. 2 h after the beginning of the surgery T3. Postoperatively T4. 24 h post-operative T5. 72 h post-operative	Colorectal cancer (30), liver cancer (46)	-	-	-	Flow cytometry	NK cell number
Cui et al. [24]	oxycodone = 57.72 ± 4.63 morphine = 58.63 ± 5.56	T1. Before injection T2. 30 min after injection T3. 6 h after injection T4. 12 h after injection T5. 24 h after injection	-	-	-	-	Flow cytometry	NK cell number
Wan et al. [25]	OF = 68.4 ± 8.5 SF = 65.7 ± 7.9	T1.30 min preoperatively T2. 12 h post-operative T3. 24 h post-operative T4. 48 h post-operative T5. 72 h post-operative	Colon cancer (87), rectal cancer (46)	Stage I (45), Stage II (69), Stage III (19)	-	Laparoscopic	Standard chromium-51 release assay	NK cell number
Sutic et al. [26]	-	T1. Preoperatively T2. 1 day post-operative T3. 6 days post-operative	-	-	-	-	Flow cytometry	NK cell number
Kim et al. [27]	Opioid group = 66 ± 9 ON-Q group = 63 ± 11	T1. Preoperatively T2. 24 h post-operative T3.48 h post-operative	Colorectal cancer (69)	Stage I (14), Stage II (9), Stage III (30), Stage IV (2)	-	Anterior resection (23), low anterior resection (18), left hemicolectomy (10), right hemicolectomy (8)	LDH assay	NK cell cytotoxicity
Beilin et al. [28]	Small dose of Fentanyl (SDFA) = 40–66 Large dose of fentanyl (LDFA) = 45–60	T1. During premeditation was given T2. 60 min after beginning of surgery T3. 24 h post-operative T4.48 h post-operative	Cholecystectomy (11), myomectomy (9), colon cancer (17), stomach cancer (1), uterine cancer (1), ovarian cancer (1)	-	-	-	Standard chromium-51 release assay	NK cell cytotoxicity
Mathiesen et al. [29]	Not transfused = 69 (25–90) transfused = 74(37–90)	-	-	-	-	-	Flow cytometry	NK cell cytotoxicity
Tartter et al. [30]	No transfusion = 66 Transfused = 66	-	-	-	-	Right hemicolectomy (23), transverse colectomy (4), left hemicolectomy (12), sigmoid resection (24), interior resection (34), abdominoperineal resection (15), subtotal colectomy (4)	Standard chromium-51 release assay	NK cell cytotoxicity
Mathiesen et al. [31]	No previous transfusion = 67 ± 12.3 Previous transfusion = 72 ± 9.8	T1.1 day before operation T2.6 months post-op	-	-	-	-	Flow cytometry	NK cell cytotoxicity
Qiu et al. [32]	Non-transfused = 58.15 ± 12.38 Transfused = 58.06 ± 12.23	T1. Before blood transfusion T2.2 days after blood transfusion	-	-	-	Laparoscopic	-	NK cell number
Heiss et al. [33]	Autologous blood transfusion = 63 Allogenic blood transfusion = 61 No blood transfusion = 62	T1. 11 days prior to surgery T2. Prior to surgery T3. 3 days postoperatively T4. 8 days postoperatively	Rectum cancer (11), colon cancer (11)	-	Rectum (11), colon (11)	Anterior resection (11), sigma resection (3), left hemicolectomy (3), right hemicolectomy (5)	Bis-carboxyethyl-carboxyfluorescein (BCECF)	NK cell cytotoxicity
Flodgren et al. [34]	-	-	-	-	-	-	Standard chromium-51 release assay	NK cell cytotoxicity
Liu et al. [35]	-	-	-	-	-	-	Flow cytometry	NK cell activity and cytotoxicity

Seven studies [21, 22, 25, 29, 30, 32, 33] **(**Table 1**)** stated the type of operations included in the study.

### Blood collection and NK cell analysis methods in the included studies

Across nine studies that investigated pharmaceutical interventions in CRC patients [20–28] **(**Table 1**)**, bloods were collected at a range of timepoints from pre-operative sampling up to 72 h postoperatively. Two studies [23, 25] collected serum and 10 studies [20–22, 26–29, 31, 33, 35] collected PBMCs. Four studies [24, 30, 32, 34] did not identify the blood fraction analysed.

There are numerous analytical techniques available to characterise NK cells which include total number, cytotoxicity and activity of NK cells. Four studies [25, 28, 30, 34] **(**Table 1**)** used the standard chromium-51(^51^Cr) release assay, the “gold standard” to assess the cytotoxicity of NK cells [36]. Five studies [20, 21, 23, 24, 26] used flow cytometry to quantify the number of NK cells by surface CD56 and CD16 staining, whereas three studies [29, 31, 35] used flow cytometry to assess the NK cytotoxicity **(**Table 1**)**. One study [22] analysed NK cell cytotoxicity by measuring interferon-γ release. Lastly, one study [27] used the lactate dehydrogenase (LDH) assay to measure the NK cell cytotoxicity.

### Influence of pharmaceutical interventions on NK cell cytotoxicity and number

Nine studies [20–28] investigated the impact of perioperative anaesthetic and analgesics on NK cell cytotoxicity and number (Table 2) including the influence of medications on NK cell function for CRC patients undergoing surgery.

**Table 2:** Study quality and effects of perioperative intervention drugs in colorectal cancer patient studies

First author	Year	Country	Rationale	JBI score	Total, n	Control	Cases	Control drug	Case drug (dosage)	Findings
Li et al. [20]	2022	China	Effect of dexmedetomidine with etomidate on cellular immune function in radical rectal cancer resection	7/9	100	-	-	Dexmedetomidine	Dexmedetomidine + etomidate (1 μg/kg)	NK cells showed a trend increase in both groups postoperatively (8.45 ± 2.93; 11.12 ± 4.34) compared to preoperatively (5.66 ± 2.93; 5.53 ± 1.24)
Oh et al. [21]	2022	Korea	Effect of propofol and sevoflurane-based anaesthesia during CRC surgery on NK cells	12/13	153	76	77	Propofol	Sevoflurane	NK cells decrease in both groups 1 h postoperatively compared to preoperatively (17.9 ± 12.7 vs. 20.4 ± 13.4; 20.7 ± 11.9 vs. 20.8 ± 11.3), but has a trend increased in 24 h postoperatively compared to 1 h (18.6 ± 11.6 vs. 17.9 ± 12.7; 21.3 ± 10.8 vs. 20.7 ± 11.9)
Cho et al. [22]	2021	Korea	Effect of subanaesthetic-dose ketamine on NK cell activity in CRC surgery	12/13	100	50	50	Saline (0.9%) + propofol (1.5-2 mg/kg^−1^) + remifentanil (1 μg/kg^−1^)	Ketamine (0.05 mg.kg^−1^ h^−1^) + propofol (1.5-2 mg/kg^−1^) + remifentanil (1 μg/kg^−1^))	NK cell cytotoxicity decreased significantly in 1 h (52.4%; 53.2%), 24 h (49.9%; 51.1.%) and 48 h (43.6%; 45.5%) postoperatively compared to preoperatively in both groups
Song et al. [23]	2017	China	Effect of different anaesthesia and analgesia methods on immune function with patients undergoing tumour resection surgery	7/9	76	38	38	Propofol (3–4 μg /mL) + fentanyl (2-4 μg/kg) + cisatracurium (0.02 mg/kg) + remifentanil (0.1–0.2 μg /kg/min) Analgesia (Intravenous): sufentanil (1 μg /mL)	Propofol (3-4 μg/mL) + fentanyl (2-4 μg/kg) + lidocaine (1%) + ropivacaine (0.375%) Analgesia (Epidural): sufentanil (0.2 μg /mL)	No significant decrease in NK cell number between two groups at any timepoint: 30 min before anaesthesia (T1) (17.32 ± 3.35; 16.73 ± 3.54) 2 h after the beginning of the surgery (T2) (19.35 ± 3.54; 18.86 ± 3.31) immediately after surgery (T3) (19.84 ± 3.47; 19.53 ± 3.46) 24 h after surgery (T4) (18.27 ± 3.7; 17.76 ± 3.63) 72 h after surgery (T5) (16.49 ± 4.83; 16.73 ± 3.56)
Cui et al. [24]	2017	China	Effect of oxycodone hydrochloride injection on the immune function in radical resection	8/9	80	40	40	Oxycodone (5 mg)	Morphine (5 mg)	NK cells were significantly decreased in both groups from different timepoint compared baseline: before injection (T0) (30.6 ± 3.8; 31.6 ± 3.5) 0.5 h after injection (T1) (28.6 ± 3.1; 26.4 ± 3.2) 6 h after injection (T2) (23.4 ± 2.8; 20.2 ± 2.8) 12 h after injection (T3) (25.6 ± 2.2; 23.6 ± 3.2) 24 h after injection (T4) (29.3 ± 2.3; 26.4 ± 2.7)
Wan et al. [25]	2020	China	Effect of oxycodone combined with flurbiprofen axetil on post-operative analgesia and immune function in radical resection of CRC	12/13	133	65	68	Flurbiprofen (3 mg/kg) + oxycodone (0.1 mg/kg) (OF group)	Flurbiprofen (3 mg/kg) + sufentanil (0.1 μg/kg) (SF Group)	NK T cells were decreased in both groups compared to preoperatively, but NK cells were higher in the OF group at 48 and 72 h postoperatively
Sutic et al. [26]	2011	Croatia	Effect of two different post-operative pain management techniques on innate immunity in CRC surgery	5/9	80	-	-	Epidural Analgesia	Intravenous Analgesia	Epidural analgesia shows that significant increase in NK and NKT cells compare to patients who received intravenous analgesia
Kim et al. [27]	2016	Korea	Effect of an opioid-based analgesic regimen versus a local anaesthetic wound infiltration-based analgesic regimen on immune modulation in patients undergoing laparoscopic resection of CRC	8/13	59	30	29	Propofol + fentanyl (2000 μg)	Propofol + ropivacaine (0.5%) + tramadol (450 mg)	NK cells were decreased in both group 24 h postoperatively but recovered 48 h postoperatively
Beilin et al. [28]	1996	Israel	Effect of opiate dosage during anaesthesia on immunosuppression observed in the post-operative period	7/9	40	20	20	Small dose of fentanyl (SDFA) (1 μg/kg)	Large dose of fentanyl (LDFA) (75–100 μg/kg)	Significant decrease in NK cell cytotoxicity with SDFA and LDFA after 24 h postoperatively compared to before injection (22.7 ± 2.71 vs. 36.7 ± 2.43); (20.7 ± 2.21 vs. 40.5 ± 2.36). By 48 h, NK cells in SDFA recovered to control values, but LDFA still remains significantly low (34.7 ± 1.41; 27.6 ± 1.85)

### Effect of general anaesthetics on NK cells

Anaesthetic medications modulate the function of immune cells both directly and indirectly. In one study, the commonly used a_2-_adrenoreceptor agonist dexmedetomidine was used alone or combined with etomidate, which is used to relieve restlessness when a patient is awakening from general anaesthetic [20]. NK cell numbers significantly increased (*p* < 0.01) postoperatively compared to preoperatively in with dexmedetomidine + etomidate (pre-op,5.66±2.93; post-op,8.45±2.93) and non-significantly (*p* = 0.634) with dexmedetomidine (pre-op,5.53±1.24; post-op,11.12 ± 4.34)[20]. Oh et al*.*[21] reported that the fraction of circulating NK cell numbers was not significantly different between the propofol and sevoflurane groups (pre-op,20.4±13.4% vs. 20.8±11.3%, *p* = 0.863; 1 h post-op,17.9±12.7% vs. 20.7±11.9%, *p* = 0.136; 24 h post-op,18.6±11.6% vs. 21.3±10.8%, *p* = 0.151; respectively). In addition, Cho et al. [22] found that NK cell number decreased at 1 h, 24 h and 48 h postoperatively compared to baseline in both patient groups treated with propofol + remifentanil (pre-op,2.36; 1 h post-op,1.12; 24 h post-op,1.10; 48 h post-op,1.21) and also the additional administration of ketamine (pre-op,2.45; 1 h post-op,1.16; 24 h post-op 24 h,1.21; 48 h post-op,1.48) [22]. These studies have shown that different combinations of anaesthetic medications have different effects on NK cells.

### Effect of combined general anaesthetic and epidural local anaesthetics on NK cells

General anaesthetic is commonly used in combination with epidural anaesthetic medications in operative settings. Song et al. [23] assessed the impact on NK cell numbers when propofol was used as the general anaesthetic medication with combinations of cisatracurium + remifentanil (control group) or lidocaine + ropivacaine (observe group). There was no significant difference between the control and observe groups at any of the timepoints (30 min before anaesthesia: 7.32±3.35 vs. 16.73±3.54, *p* = 0.457; 2 h pre-op: 19.35±3.54 vs. 18.86±3.31, *p* = 0.535; immediately after surgery: 19.84±3.47 vs. 19.53±3.46, *p* = 0.698; 24 h post-op: 18.27±3.75 vs. 17.76±3.63, *p* = 0.549; 72 h post-op: 16.49±4.83 vs. 16.73±3.56, *p* = 0.805; respectively) [23]. Unfortunately, this study did not compare within group differences between the timepoints. Overall, the number of NK cells was not significantly different between the different combinations of general and local anaesthetic medications.

### Effect of analgesics

Analgesics are used to reduce surgical stress and post-operative complications. Cui et al. [24] found the administration of oxycodone and morphine after the surgery to patients with the combination of general anaesthesia showed an effect on the NK cell number. The number of NK cells was significantly lower after the injection of oxycodone and morphine compared to before injecting these medications; before injection (30.6±3.8 vs. 31.6 ± 3.5), 0.5 h (28.6 ± 3.1 vs. 26.4 ± 3.2), 6 h after injection (23.4 ± 2.8 vs. 20.2 ± 2.8), 12 h after injection (25.6 ± 2.2 vs. 23.6 ± 3.2) and 24 h after injection (29.3 ± 2.3 vs. 26.4 ± 2.7). However, NK cell numbers were higher in the oxycodone group than in patients who received morphine at corresponding timepoints. In addition, Wan et al. [25] reported that the combination oxycodone + flurbiprofen versus sufentanil + flurbiprofen that were given to patients after the surgery resulted in lower number of NK cells in 12 h, 24 h, 48 h and 72 h postoperatively compared to 30 min pre-surgery, but the number of NK cells was comparatively higher at 48 h and 72 h postoperatively in the oxycodone + flurbiprofen group; however, statistical values were not provided [25]. Furthermore, Sutic et al. [26] compared the effect of epidural analgesic compared to intravenous analgesic on NK cell numbers, showing that patients who received the intravenous analgesic had significantly lower NK cell numbers compared to those with epidural analgesic unfortunately, and no statistical values were provided. Similarly, another study [27] looked at the effects of intravenous analgesic in combination with local anaesthetics, fentanyl versus fentanyl + ropivacaine + tramadol. Patients in both groups showed a decrease in NK cell cytotoxicity 24 h postoperatively compared to preoperatively, and the NK cell cytotoxicity recovered slowly over 48 h in both groups [27], similarly no statistical values were provided. These studies have shown that analgesic medication may have a transient influence on the number and cytotoxicity of the NK cells.

### Analgesic dose; response effect on NK cells.

To examine dose effects of analgesics on NK cell cytotoxicity, one study assessed different doses of fentanyl (1 μg/kg vs. 75–100 μg/kg) administered throughout the surgery [28]. NK cell cytotoxicity was found to be significantly decreased in both groups with different doses of fentanyl at 24 h post-operation compared to pre-operative levels (small dose: 36.7%±2.43% to 22.7%±2.71%; large dose: 40.5%±2.36% to 20.7%±22.21%). By 48 h post-operation, NK cell cytotoxicity almost completely recovered in patients receiving lower doses of fentanyl (34.7%±1.41%), although they remained significantly lower in patients receiving larger doses (27.6%±1.85%). This highlights that the dose of analgesics plays a role in affecting the cytotoxicity of NK cells in CRC patients. Overall, NK cell number and/or cytotoxicity were affected in patients following all forms of perioperative interventions, supporting the hypothesis that post-surgical immune suppression inhibits the capacity of the immune system to eliminate remaining tumour cells.

### Influence of blood transfusions on NK cell cytotoxicity and numbers

Five included studies [29–33] analysed the number and cytotoxicity of NK cells for CRC patients who received a blood transfusion (Table 4) of supplementary material.

### Whole blood transfusion on NK cell cytotoxicity.

Three studies investigated NK cell cytotoxicity in CRC patients who received a whole blood transfusion compared to patients without transfusion [29–31]. Two of these studies [29, 30] reported a 30% decrease in NK cell cytotoxicity associated with transfusion of whole blood products. Another study found no difference in NK cell cytotoxicity between patients who received a whole blood transfusion versus patients who received leucocyte-depleted (red blood cell and platelet unit) blood products [31] (Table 4) of supplementary material .

Allogenic and autologous blood transfusion in relation to NK cell number and cytotoxicity.

Two studies [32, 33] investigated the NK cell number and cytotoxicity in CRC patients who received allogenic and autologous blood transfusions (Table 4) of supplementary material. Qiu et al. [32] found that NK cell number was decreased after allogeneic blood transfusion when compared to pre-transfusion levels (sporadic CRC: 13.4 ± 3.25 to 8.16 ± 0.56; hereditary CRC: 13.28 ± 0.3.46 to 8.39 ± 1.93). A similar study by Heiss et al. [33] investigating NK cell cytotoxicity pre- and postoperatively reported a similar decrease in cytotoxic capacity in post-operative patients who received allogenic blood transfusion (pre-op: 63.8; 3d post-op: 42.3; 8d post-op: 31.9) compared to those with no transfusion (pre-op: 52.1; 3d post-op: 32.5; 8d post-op: 23.5). In contrast, patients who received autologous blood transfusion had a non-significant increase on the 8th post-operative day in cytotoxicity of NK cells compared with pre-operative levels (pre-op: 37.4; 3d post-op: 40.8; 8d post-op: 53.9). These studies suggest that, in addition to considering the impact of pharmaceuticals on CRC recurrence and metastasis in the operative environment, the impact on different type of blood transfusion also requires further study and consideration.

### Influence of perioperative medications on NK cell cytotoxicity and activity *in vitro* conditions

To further understand the mechanistic impact medications may have on patient immunity perioperatively, two studies [34, 35] explored the cytotoxicity of NK cells exposed to medications under *in vitro* conditions (Table 5) of supplementary material. One study [34] investigated the use of indomethacin, a non-steroidal anti-inflammatory drug (NSAID) compared to cimetidine, a H2 antagonist and cell culture media. Incubation with indomethacin demonstrated a significant increase in NK cell activity compared to pre-incubation (CRC patient: 17.4 ± 2.6 to 18.4 ± 2.0), whereas cimetidine had no effect compared to pre-incubation with healthy volunteers but significant increase with CRC patients (healthy volunteers: 16.7 ± 4.6 to 22.6 ± 6.5, CRC patients: 17.4 ± 2.6 to 19.5 ± 2.7) [34]. In addition, Liu et al*.* [35] reported that NK cells incubated with propofol has enhanced the killing effect of NK cells on colon cancer cells compared to the negative control group and also increased the proliferation ability of NK cells, unfortunately no statistical values were provided. This study suggests that propofol may have benefits for NK activity in operative settings. Together, these data suggest propofol and indomethacin increase NK cell activity and may be beneficial to patients that have undergone surgery.

## Discussion

Over the past 80 years, factors that result in immunosuppression, likely contributing to CRC recurrence and metastasis following CRC surgery have been studied. Studies have shown that NK cell function, but not cell number, can decline with increasing age [37], and NK cell activity is suppressed in cases of increased tumour burden [38] or metastasis in animal studies [39]. NK cell killing of target cells is dependent on the activation and inhibition of specific receptors and there is an age-related decline in the percentage of NK cells expressing the activation receptors (NKP46 or NKP30) required for effective NK cell killing of target cells and an increase in the expression of the inhibitory receptor, KLRG1 [40, 41]. These considerations likely have important implications in the context of CRC surgery, given the average age range of patients. Even though surgery is the mainstay for solid tumours, surgery itself is thought to promote metastasis by releasing tumour cells into the circulation and by suppressing the immune system. Despite the beneficial effects of surgical removal of the primary tumour, studies have shown that NK cell activity is suppressed due to both the surgery-induced stress response and anaesthetic-induced immunosuppression [42], and this suppression can last for several days depending on the degree surgical trauma. Importantly, it is well established that circulating tumour cells are present at the time of surgical resection [43], and these cells may metastasise to distal organs [44]. Whether or not the loss of NK cell activity during the surgical period facilitates the escape and metastasis of these tumour cells is currently unclear but may be one of the factors that contribute to cancer recurrence. Studies across various types of cancer [14, 45, 46] including colorectal, pancreatic and breast cancer have demonstrated a significant post-operative decrease in NK cell numbers. However, the surgical impact on NK cells appears to be transient, with numbers gradually returning to pre-operative baseline by post-operative day 30. The multifactorial nature of NK cell modulation following surgery [47] necessitates investigation into the recovery kinetics of NK cell function and activity within a specific post-operative timeframe. This systematic review primarily focused on examining the number and function of NK cells following CRC patients with perioperative pharmaceutical interventions and blood transfusions.

Of the different perioperative medication combinations examined, most studies investigated the effect of anaesthetic and analgesic medications on the function of immune cells. Opioids such as oxycodone, fentanyl and morphine decrease the cytotoxicity of NK cells against tumour cell lines [24–26, 28]. Opioids can affect cell-mediated and humoral immunity by acting directly on immune cells and through sympathetic activity [48]. This has been shown to promote angiogenesis and lymphangiogenesis, which could lead to an increase both in tumour growth and survival [49]. Opioids also produce alterations in the function of cells involved in surveillance, including macrophages and NK cells [50, 51]. Opioids have a direct effect on immune function acting through the opioid receptors expressed on immune cells including NK cells [52]. Opioids can also reduce the primary function of NK cells by suppressing the cytolytic function of circulating lymphocytes [49]. Studies have shown that morphine suppresses the production of interferons leading to a decrease in NK cell cytotoxicity [51, 53]. Morphine can act on the periaqueductal grey and sympathetic nervous system to increase neuropeptide Y release, acting on the peripheral Y1 receptor to inhibit NK cell cytotoxicity [54, 55]. Fentanyl is commonly used for CRC surgery patients, and there is evidence of dose-related effects on the cellular immune response in humans [28, 56]. Given that NK cell cytotoxicity and number were decreased with opioid (e.g. morphine, fentanyl), the literature suggests that these medications may have negative outcomes for CRC patients. Determining the appropriate dose is crucial for balancing effective pain relief with minimising adverse effects and the risk of dependence.

NSAIDS and opioids are commonly used to treat pain in surgical patients, with diverse immunomodulatory effects. NSAIDs directly act on prostaglandin release by inhibiting the COX1 and COX2 enzymes in the CNS which in turn inhibits prostaglandin synthesis [57]. Murine model [58] has shown that NSAIDS increases the cytotoxicity of NK cells by downregulating the MHC-I expression which highlights the anti-tumour immune responsiveness of NSAIDs. In contrast, opioids have a less immediate effect but are potent in their reaction for relieving acute pain. They bind to opioid receptors in the brain which reduces pain signalling in the body [59]. However, prolonged use of opioids increases activity of the HPA axis, thereby increasing the release of immunosuppressive glucocorticoids which leads to a decrease in the function of NK cells [60].

Anaesthetics have been shown to have varied influence on the immune system. For instance, while ketamine reduces NK cell activity and increases breast cancer metastasis in animal models, propofol did not suppress NK cell cytotoxicity [61]. In the in vitro study, propofol shown to enhance the killing effect of NK cells by increasing the level of activated receptors and decreased level of the inhibitory receptors [35]. Lim et al*.* [62] and Jaura et al*.* [63] also revealed that propofol regulates the metastasis and apoptosis of tumour cells to a certain extent in vitro model which suggest that propofol could improve the activity of NK cell. In addition, dexmedetomidine with or without etomidate has been shown to preserve the function of NK cells [20, 64]. Dexmedetomidine acts via the central and peripheral activation of α2-adrenoceptors that inhibit the release of catecholamines and sympathetic activity [65, 66]. A reduction in catecholamines can reduce the activity of the hypothalamic-pituitary (HPA) axis [67, 68]. Under surgical stress, there is an increase in cortisol, mediated by the HPA axis, which attenuates NK cell cytotoxicity [60]. However, given that dexmedetomidine reduces the activity of the HPA axis and thus the production of cortisol, NK cell function is preserved. Cumulatively this could lead to increased immune response and better overall outcomes [69]. Epidural anaesthesia, when combined with general anaesthesia, showed an increase in NK cell number [26]. This combination was also found to mitigate the surgical stress-related impairment of anti-tumour response, fasten intestinal function and improve long-term outcomes [70, 71]. This suggests that epidural anaesthesia could increase NK cell cytotoxicity and potentially decrease recurrence and metastases.

Patients presenting for major surgery are frequently anaemic in the pre-operative period [72, 73], possibly due to blood loss in the gastrointestinal tract from their CRC. Blood transfusions have been shown to suppress cytotoxic cell and monocyte activity [32, 74]. Separately, transfusions are also associated with cancer recurrence and metastasis [75]. Furthermore, in gastric cancer patients, blood transfusion decreases the number of NK cells and activity, decreases phagocytic activity and also affecting delayed-type hypersensitivity responses [76]. Patients’ immunity may thus be impaired by both surgical trauma and blood transfusion [77], increasing cancer-related mortality and recurrence [75, 78]. Allogenic blood transfusions show a decrease in cell-mediated immunity [79] and the number of immune cells compared to autologous transfusions in gastric cancer patients [80], suggesting that autologous blood transfusions may be preferable. Clinically, patients requiring transfusion have higher ratio for cancer recurrence [75]. This may be due to the introduction of foreign immune cells and alterations in NK cell number and/or function. Unfortunately, there are no studies that directly assess whether the context leading to a transfusion, or the process of transfusion itself affect NK cells.

In in vitro studies, indomethacin and propofol have shown to enhance the killing effect of NK cells. Indomethacin is a NSAID that inhibits the synthesis of prostaglandins, critical mediators of inflammation that produce COX enzymes [81]. Prostaglandins can suppress NK cell activity, so by reducing the prostaglandin levels, indomethacin likely restores cellular immunity by increasing NK cell activity [81]. In contrast, propofol works by inhibiting the function of neurotransmitter gamma-aminobutyric acid (GABA) through GABA-A receptors [82] and reduces the PGE2 production in vitro, a known mediator of pain and inflammation shown to inhibit NK cell cytotoxicity [83, 84]. Previous studies have also shown that propofol has potential anti-tumour activity [85]. This promotes post-operative immune surveillance and inhibits the metastasis of tumours by increasing expression of NK cell activation receptors, while decreasing inhibitory receptor expression [86]. These potential mechanisms have yet to be shown in vivo, although offer potential avenues for immune modulation.

Surgical interventions and anaesthesia can lead to imbalance of the innate and adaptive immunity and impair the immune function [85]. The inhibition of immune function after surgery has a significant correlation with the degree of trauma of surgery [87]. Animal models have also demonstrated the suppression of NK cell cytotoxicity in the post-operative period can increased metastatic formation [88–90]. Unfortunately, in our review, none of the studies assessed or accounted for the effect of surgical trauma on NK cells. Despite this significant limitation, the differences observed in NK cell function with propofol and dexmedetomidine suggest that these drugs beneficially affect NK cells beyond the influence of surgical trauma.

While this study includes a comprehensive literature search strategy and broad spectrum of data collected from included studies, there are some limitations. These include the methodological heterogeneity of studies in examining NK cell numbers and cytotoxicity. This included differences in sampling methods and sample size, patients receiving different transfusions between studies, diverse pharmaceutical interventions and inconsistent approaches to data analysis. While most studies identified their aims, participant demographics and inclusion criteria for the patients, few included power calculations for the sample size. None of the studies in our review excluded any mutual interference of anaesthetic and analgesic drug. While this is a limitation of the study, the nature of the patients and interventions in these studies make it unlikely that this could be assessed outside of a preclinical animal study.

In conclusion, this systematic review provides evidence that existing cancer, metastatic disease, transfusion and pharmaceutical interventions affect NK cell number, activity and cytotoxicity, mostly in a detrimental fashion. This can be influenced by the class of drugs, the dosage and the combination of drugs. However, the existing evidence is extremely limited and varied in intervention approach, meaning further work is required to identify direct influences and minimise the impact that perioperative interventions have on NK cell numbers and function.

## Supplementary Information

Below is the link to the electronic supplementary material.

Supplementary file1 (DOCX 55 KB)

Supplementary file2 (DOCX 24KB)

## Data Availability

No datasets were generated or analysed during the current study.
