# Supplementary material for: Interventional factors influencing natural killer cell immunity in colorectal cancer: a systematic review
Source: Cancer Immunol Immunother. 2025 Feb 1;74(3):91. doi: 10.1007/s00262-024-03900-5 (PMC11787105; doi:10.1007/s00262-024-03900-5)
Supplement: Supplementary file 1 — Supplementary file1 (DOCX 55 KB) [file 262_2024_3900_MOESM1_ESM.docx]

**Supplementary Materials**

**Supplementary Table S1**. Search Strategy

| **Search #** | **Search Strategy** |
| --- | --- |
| 1 | exp Killer Cells, Natural/ |
| 2 | (cell* adj1 (natural killer or NK or natural k)).ti,ab,kw,kf. |
| 3 | Immunity, Innate/ |
| 4 | (innate lymphoid cell* or ILC or ILC1).ti,ab,kw,kf. |
| 5 | Dendritic Cells/ |
| 6 | immune cell*.ti,ab,kw,kf. |
| 7 | exp T-Lymphocytes/ |
| 8 | (T-cell* or T-Lymphocyte*).ti,ab,kw,kf. |
| 9 | Macrophages/ |
| 10 | macrophage*.ti,ab,kw,kf. |
| 11 | 1 or 2 or 3 or 4 or 5 or 6 or 7 or 8 or 9 or 10 |
| 12 | exp Colorectal Neoplasms/ |
| 13 | ((colorectal or colon or colonic or rectum or rectal or bowel) adj3 (neoplas* or cancer* or tumo?r* or carcinoma* or adenocarcinoma*)).ti,ab,kw,kf. |
| 14 | 12 or 13 |
| 15 | exp Anesthetics/ |
| 16 | an?esthetic*.ti,ab,kw,kf. |
| 17 | (propofol or halothane or etomidate or ketamine or midazolam or thiopental or methoxyflurane or desflurane or isoflurane or sevoflurane or barbiturate* or benzodiazepine* or dexmedetomidine).af. |
| 18 | exp Neuromuscular Blocking Agents |
| 19 | ((neuromuscular or neuro-muscular) adj blocker*).ti,ab,kw,kf. |
| 20 | exp Adrenergic agents/ |
| 21 | (alpha* adj2 agonist*).ti,ab,kw,kf. |
| 22 | (beta adj2 block*).ti,ab,kw,kf. |
| 23 | exp Cholinergic Antagonists/ |
| 24 | (anticholinergic* or anti-cholinergic* or cholinergic antagonist*).ti,ab,kw,kf. |
| 25 | (bupivacaine or levobupivacaine).af. |
| 26 | exp Analgesics/ |
| 27 | Analgesia, epidural/ |
| 28 | (lidocaine or lignocaine or ropivacaine).af. |
| 29 | analgesi*.ti,ab,kw,kf. |
| 30 | NSAID*.ti,ab,kw,kf. |
| 31 | ((non steroid* or nonsteroid*) adj (anti-inflammator* or antiinflammator*)).ti,ab,kw,kf. |
| 32 | exp Anti-Infective Agents/ |
| 33 | (antibiotic* or anti-biotic* or anti-bacterial or antibacterial or anti-infective anti-inflammator*).ti,ab,kw,kf. |
| 34 | exp Serotonin Antagonists/ |
| 35 | ((5-HT3 or 5HT3) adj2 receptor).ti,ab,kw,kf. |
| 36 | serotonin antagonist*.ti,ab,kw,kf. |
| 37 | exp Cyclooxygenase inhibitors/ |
| 38 | (cox adj2 inhibitor*).ti,ab,kw,kf. |
| 39 | (acetylsalicylic acid or indomethacin or celecoxib).af. |
| 40 | exp Dopamine Antagonists/ |
| 41 | (dopamine adj2 antagonist*).ti,ab,kw,kf. |
| 42 | exp Neurokinin-1 Receptor Antagonists/ |
| 43 | neurokinin* receptor.ti,ab,kw,kf. |
| 44 | exp Morpholines/ |
| 45 | exp Glucocorticoids/ |
| 46 | glucocorticoid*.ti,ab,kw,kf. |
| 47 | dexamethasone.af. |
| 48 | (cortisone* or corticosteroid*).ti,ab,kw,kf. |
| 49 | exp Anticoagulants/ |
| 50 | (anticoagulant* or anti-coagulant* or heparin*).ti,ab,kw,kf. |
| 51 | (apixaban or rivaroxaban or dabigatran or dalteparin or enoxaparin).af. |
| 52 | opioid*.ti,ab,kw,kf. |
| 53 | (morphine or oxycodone or tramadol or fentanyl or hydromorphone or remifentanil or sufentanil or pethidine or methadone or buprenorphine).af. |
| 54 | Melatonin/ |
| 55 | melatonin.ti,ab,kw,kf. |
| 56 | exp Proton Pump Inhibitors/ |
| 57 | proton pump inhibitor*.ti,ab,kw,kf. |
| 58 | exp Antiemetics/ |
| 59 | antiemetic*.ti,ab,kw,kf. |
| 60 | exp Cannabinoids/ |
| 61 | Cannabis/ |
| 62 | (cannabis or cannabinoid* or cannabigerol* or tetrahydrocannabinol* or cannabidiol* or dronabinol* or THC or nabiximol*).ti,ab,kw,kf. |
| 63 | Medical Marijuana/ |
| 64 | marijuana.ti,ab,kw,kf. |
| 65 | exp Blood Transfusion/ |
| 66 | transfus*.ti,ab,kw,kf. |
| 67 | 15 or 16 or 17 or 18 or 19 or 20 or 21 or 22 or 23 or 24 or 25 or 26 or 27 or 28 or 29 or 30 or 31 or 32 or 33 or 34 or 35 or 36 or 37 or 38 or 39 or 40 or 41 or 42 or 43 or 44 or 45 or 46 or 47 or 48 or 49 or 50 or 51 or 52 or 53 or 54 or 55 or 56 or 57 or 58 or 59 or 60 or 61 or 62 or 63 or 64 or 65 or 66 (3983378) |
| 68 | 11 and 14 and 67 |
| 69 | limit 68 to english language |
| 70 | limit 69 to (comment or editorial or letter or news) |
| 71 | 69 not 70 |

**Supplementary Table S2** The Preferred Reporting Items for Systematic Reviews and Meta-Analyses (PRISMA) Checklist

| **Section and Topic** | **Item #** | **Checklist item** | **Location where item is reported** |
| --- | --- | --- | --- |
| **TITLE** | | |  |
| Title | 1 | Identify the report as a systematic review. | 1-2 |
| **ABSTRACT** | | |  |
| Abstract | 2 | See the PRISMA 2020 for Abstracts checklist. | 40 |
| **INTRODUCTION** | | |  |
| Rationale | 3 | Describe the rationale for the review in the context of existing knowledge. | 86-92 |
| Objectives | 4 | Provide an explicit statement of the objective(s) or question(s) the review addresses. | 97-99 |
| **METHODS** | | |  |
| Eligibility criteria | 5 | Specify the inclusion and exclusion criteria for the review and how studies were grouped for the syntheses. | 143-150 |
| Information sources | 6 | Specify all databases, registers, websites, organisations, reference lists and other sources searched or consulted to identify studies. Specify the date when each source was last searched or consulted. | 133-134 |
| Search strategy | 7 | Present the full search strategies for all databases, registers and websites, including any filters and limits used. | 135-140 & Supplementary Table S1 |
| Selection process | 8 | Specify the methods used to decide whether a study met the inclusion criteria of the review, including how many reviewers screened each record and each report retrieved, whether they worked independently, and if applicable, details of automation tools used in the process. | 151-154 |
| Data collection process | 9 | Specify the methods used to collect data from reports, including how many reviewers collected data from each report, whether they worked independently, any processes for obtaining or confirming data from study investigators, and if applicable, details of automation tools used in the process. | 166-170 |
| Data items | 10a | List and define all outcomes for which data were sought. Specify whether all results that were compatible with each outcome domain in each study were sought (e.g. for all measures, time points, analyses), and if not, the methods used to decide which results to collect. | 166-170 |
|  | 10b | List and define all other variables for which data were sought (e.g. participant and intervention characteristics, funding sources). Describe any assumptions made about any missing or unclear information. | 170 |
| Study risk of bias assessment | 11 | Specify the methods used to assess risk of bias in the included studies, including details of the tool(s) used, how many reviewers assessed each study and whether they worked independently, and if applicable, details of automation tools used in the process. | 157-163 & Supplementary Table S3 |
| Effect measures | 12 | Specify for each outcome the effect measure(s) (e.g. risk ratio, mean difference) used in the synthesis or presentation of results. | N/A |
| Synthesis methods | 13a | Describe the processes used to decide which studies were eligible for each synthesis (e.g. tabulating the study intervention characteristics and comparing against the planned groups for each synthesis (item #5)). | N/A |
|  | 13b | Describe any methods required to prepare the data for presentation or synthesis, such as handling of missing summary statistics, or data conversions. | N/A |
|  | 13c | Describe any methods used to tabulate or visually display results of individual studies and syntheses. | N/A |
|  | 13d | Describe any methods used to synthesize results and provide a rationale for the choice(s). If meta-analysis was performed, describe the model(s), method(s) to identify the presence and extent of statistical heterogeneity, and software package(s) used. | N/A |
|  | 13e | Describe any methods used to explore possible causes of heterogeneity among study results (e.g. subgroup analysis, meta-regression). | N/A |
|  | 13f | Describe any sensitivity analyses conducted to assess robustness of the synthesized results. | N/A |
| Reporting bias assessment | 14 | Describe any methods used to assess risk of bias due to missing results in a synthesis (arising from reporting biases). | N/A |
| Certainty assessment | 15 | Describe any methods used to assess certainty (or confidence) in the body of evidence for an outcome. | N/A |
| **RESULTS** | | |  |
| Study selection | 16a | Describe the results of the search and selection process, from the number of records identified in the search to the number of studies included in the review, ideally using a flow diagram. | 174-180 |
|  | 16b | Cite studies that might appear to meet the inclusion criteria, but which were excluded, and explain why they were excluded. | Figure 1 |
| Study characteristics | 17 | Cite each included study and present its characteristics. | Table 2,3&4 |
| Risk of bias in studies | 18 | Present assessments of risk of bias for each included study. | Supplementary Table 3 |
| Results of individual studies | 19 | For all outcomes, present, for each study: (a) summary statistics for each group (where appropriate) and (b) an effect estimate and its precision (e.g. confidence/credible interval), ideally using structured tables or plots. | 190-345 |
| Results of syntheses | 20a | For each synthesis, briefly summarise the characteristics and risk of bias among contributing studies. | N/A |
|  | 20b | Present results of all statistical syntheses conducted. If meta-analysis was done, present for each the summary estimate and its precision (e.g. confidence/credible interval) and measures of statistical heterogeneity. If comparing groups, describe the direction of the effect. | N/A |
|  | 20c | Present results of all investigations of possible causes of heterogeneity among study results. | N/A |
|  | 20d | Present results of all sensitivity analyses conducted to assess the robustness of the synthesized results. | N/A |
| Reporting biases | 21 | Present assessments of risk of bias due to missing results (arising from reporting biases) for each synthesis assessed. | N/A |
| Certainty of evidence | 22 | Present assessments of certainty (or confidence) in the body of evidence for each outcome assessed. | N/A |
| **DISCUSSION** | | |  |
| Discussion | 23a | Provide a general interpretation of the results in the context of other evidence. | 347-474 |
|  | 23b | Discuss any limitations of the evidence included in the review. | 476-486 |
|  | 23c | Discuss any limitations of the review processes used. | N/A |
|  | 23d | Discuss implications of the results for practice, policy, and future research. | N/A |
| **OTHER INFORMATION** | | |  |
| Registration and protocol | 24a | Provide registration information for the review, including register name and registration number, or state that the review was not registered. | 140 |
|  | 24b | Indicate where the review protocol can be accessed, or state that a protocol was not prepared. | N/A |
|  | 24c | Describe and explain any amendments to information provided at registration or in the protocol. | N/A |
| Support | 25 | Describe sources of financial or non-financial support for the review, and the role of the funders or sponsors in the review. | 37-38 |
| Competing interests | 26 | Declare any competing interests of review authors. | 21-24 |
| Availability of data, code and other materials | 27 | Report which of the following are publicly available and where they can be found: template data collection forms; data extracted from included studies; data used for all analyses; analytic code; any other materials used in the review. | N/A |

*From:*  Page MJ, McKenzie JE, Bossuyt PM, Boutron I, Hoffmann TC, Mulrow CD, et al. The PRISMA 2020 statement: an updated guideline for reporting systematic reviews. BMJ 2021;372:n71. doi: 10.1136/bmj.n71

For more information, visit: <http://www.prisma-statement.org/>

**Supplementary Table S3.** Risk of Bias Assessment According to the JBI Critical Appraisal Tools for RTCs and Quasi-experimental

| **Randomised controlled trials** | | | | | | | | |  | | | | | | |
| --- | --- | --- | --- | --- | --- | --- | --- | --- | --- | --- | --- | --- | --- | --- | --- |
| **Reference** | **Q1** | **Q2** | **Q3** | **Q4** | **Q5** | **Q6** | **Q7** | **Q8** | | **Q9** | **Q10** | **Q11** | **Q12** | **Q13** | **Score** |
| Kim *et al.* 2016 | Y | N | Y | U | N | U | Y | Y | | Y | Y | Y | Y | Y | 8/13 |
| Cho *et al.* 2021 | Y | Y | Y | Y | Y | U | Y | Y | | Y | Y | Y | Y | Y | 12/13 |
| Wan *et al.* 2020 | Y | Y | Y | Y | Y | Y | Y | NA | | Y | Y | Y | Y | Y | 12/13 |
| Oh *et al.* 2022 | Y | Y | Y | U | Y | Y | Y | Y | | Y | Y | Y | Y | Y | 12/13 |
| Heiss *et al.* 1997 | Y | NA | Y | NA | NA | U | Y | NA | | Y | Y | Y | Y | U | 7/13 |
| Mathiesen *et al.* 1997 | Y | NA | Y | NA | NA | U | Y | NA | | Y | Y | Y | Y | U | 7/13 |

Y = Yes, N = No, NA = Not Applicable, U = Unclear

**Key: Q1:** Was true randomisation used for assignment of participants to treat groups? Q2: Was allocation to treatment groups concealed? Q3: Were treatment groups similar at baseline? Q4: Were participants blind to the treatment assignment? Q5: Were those delivering treatment blind to treatment assignment? Q6: Were outcome assessors blind to treatment assignment? Q7: Were treatment groups treated identically other than the intervention of interest? Q8: Was follow up complete and if not, were differences between groups in terms of their follow up adequately described and analysed? Q9: Were participants analysed in the groups to which they were randomised? Q10: Were outcomes measured in the same way for treatment groups? Q11:Were outcomes measured in a reliable way? Q12: Was appropriate statistical analysis used? Q13: Was the trial design appropriate, and any deviations from the standard RCT design accounted for in the conduct and analysis of the trial?

| **Quasi-experimental study** | | | | | | | | | | | | | |
| --- | --- | --- | --- | --- | --- | --- | --- | --- | --- | --- | --- | --- | --- |
| **Reference** | **Q1** | **Q2** | **Q3** | **Q4** | **Q5** | **Q6** | **Q7** | **Q8** | **Q9** | **Score** |  |  |  |
| Benzion *et al*. 1996 | Y | Y | Y | NA | Y | NA | Y | Y | Y | 7/9 |  |  |  |
| Song *et al.* 2017 | Y | Y | Y | NA | Y | NA | Y | Y | Y | 7/9 |  |  |  |
| Cui *et al.* 2017 | Y | Y | Y | Y | Y | NA | Y | Y | Y | 8/9 |  |  |  |
| Li *et al.* 2022 | Y | U | Y | Y | Y | NA | Y | Y | Y | 7/9 |  |  |  |
| Sutic *et al.* 2011 | U | Y | Y | Y | Y | NA | Y | U | U | 5/9 |  |  |  |
| Mathiesen *et al.* 1994 | U | Y | Y | Y | N | NA | Y | Y | Y | 6/9 |  |  |  |
| Tartter *et al.* 1989 | Y | Y | NA | Y | NA | NA | Y | Y | U | 5/9 |  |  |  |
| Qiu *et al*. 2016 | Y | Y | Y | Y | Y | Y | Y | Y | Y | 9/9 |  |  |  |
| Liu *et al.* 2018 | Y | Y | Y | Y | Y | NA | Y | Y | Y | 8/9 |  |  |  |
| Flodgren *et al.* 1985 | Y | Y | Y | Y | N | N | Y | U | U | 5/9 |  |  |  |

Y = Yes, N = No, NA = Not Applicable, U = Unclear

**Key:** Q1: Is it clear in the study what is the ‘cause’ and what is the ‘effect’? Q2: Were the participants included in any comparisons similar? Q3: Were the participants included in any comparisons receiving similar treatment/care, other than the exposure or intervention of interest? Q4: Was there a control group? Q5: Were there multiple measurements of the outcome both pre and post the intervention/exposure? Q6: Was follow up complete and if not, were differences between groups in terms of their follow up adequately described and analysed? Q7: Were the outcomes of participants included in any comparisons measured in the same way? Q8: Were the outcomes measured in a reliable way? Q9: Was appropriate statistical analysis used?

**Abbreviations used in this paper:**

| **CRC** | Colorectal Cancer |
| --- | --- |
| **HPA**  **JBI**  **NK cell**  **PBMCs**  **Pre-op**  **Post-op**  **RCT** | Hypothalamic-pituitary axis  Joanna Briggs Institute  Natural Killer Cell  Peripheral Blood Mononuclear Cells  Pre-operatively  Post-operatively  Randomised Control Trials |
|  |  |
|  |  |
