# Supplementary material for: Interventional factors influencing natural killer cell immunity in colorectal cancer: a systematic review
Source: Cancer Immunol Immunother. 2025 Feb 1;74(3):91. doi: 10.1007/s00262-024-03900-5 (PMC11787105; doi:10.1007/s00262-024-03900-5)
Supplement: Supplementary file 2 — Supplementary file2 (DOCX 24KB) [file 262_2024_3900_MOESM2_ESM.docx]

**Supplementary Table 4: Study quality and effects of blood transfusion in colorectal cancer surgery**

| First Author | Year | Country | Rationale | JBI Score | Total, n | Blood Transfusion | n | Without Blood Transfusion, n | Findings |
| --- | --- | --- | --- | --- | --- | --- | --- | --- | --- |
| Mathiesen *et al*. (29) | 1994 | Denmark | Investigate the lymphocyte subsets in CRC patients with and without previous blood transfusion | 6/9 | 153 | Whole Blood Transfusion | 42 | 111 | Significant reduction in NK cell function with transfused compared to non-transfused patients |
| Tartter *et al*. (30) | 1989 | USA | Investigate NK cytotoxicity with transfused and non-transfused CRC patients | 5/9 | 115 | Whole Blood Transfusion | 14 | 101 | Transfused patients were reduced by about one-third in comparison to non-transfused patients in different effector-target cell ratio  (**50:1**: non-transfused: 56±9; transfused 34±5; **25:1**: non-transfused: 54±9; transfused 33±5; **17:1**: non-transfused: 50±9; transfused 32±5; **10:1**: non-transfused: 45±8; transfused 27±5) |
| Mathiesen *et al*. (31) | 1997 | Denmark | Examine the long-term effect on cell-mediated immunity with CRC patients either transfused with leukocyte-depleted or standard buffy-coat-depleted blood products | 7/13 | 72 | Leukocyte-depleted blood products | 21 | 30 | No significant changes in NK cell function with transfused patients compared to non-transfused patients |
|  |  |  |  |  |  | Whole Blood Transfusion | 21 |  |  |
| Qiu *et al*. (32) | 2016 | China | Investigate the impacts of perioperative blood transfusion on the immune function and prognosis in CRC patients | 9/9 | 1404 | Allogenic Blood Transfusion | 803 | 601 | NK cell number statistically decreased after blood transfusion (sporadic CRC, before transfusion: 13.4±3.25 to 8.16±.56; hereditary CRC: 13.28±.3.46 to 8.39±1.93) did not have data compared between transfused and non-transfused patients |
| Heiss *et al*. (33) | 1997 | Germany | Investigate allogenic and autologous blood transfusion on cellular immune response with CRC patient | 7/13 | 22 | Allogenic Blood Transfusion | 7 | 7 | NK cell cytotoxicity declined with allogenic blood transfusion but not statistical significance (pre-op: 63.8; 3d post-op: 42.3; 8d post-op: 31.9) and patients without transfusion (pre-op: 52.1; 3d post-op: 32.5; 8d post-op: 23.5), whereas NK cell cytotoxicity showed a trend increased with autologous blood transfusion (pre-op: 37.4; 3d post-op: 40.8; 8d post-op: 53.9) |
|  |  |  |  |  |  | Autologous Blood Transfusion | 8 |  |  |

**Supplementary Table 5: Pooled data of all the in-vitro studies with CRC patients that are relative for data extraction.**

| First Author | Year | Country | Rationale | JBI Score | Total, n | Control | Colorectal Cancer | Cell Incubation with Drug | Findings |
| --- | --- | --- | --- | --- | --- | --- | --- | --- | --- |
| Flodgren *et al*. (34) | 1985 | Sweden | Effect of cimetidine and indomethacin in certain concentrations and effector-to-target cell ratios for NK cells cytolytic activities | 5/9 | 30 | 8 | 18 | Cimetidine + Indomethacin | No significant increase after incubation of Cimetidine but showed a significantly increased with incubation of Indomethacin |
| Liu *et al*. (35) | 2018 | China | Effect of propofol on the function of NK cells in killing colon cancer cells at the cellular level | 8/9 | 40 | 20 | 20 | DMSO + Propofol | Significantly higher in Propofol group for cytotoxicity and activity compared to the control group |
